# Effects of submaximal interval training on anthropometric measurements, endurance, and flexibility of lower back and hamstrings in healthy individuals with a sedentary lifestyle

**DOI:** 10.12669/pjms.41.4.10163

**Published:** 2025-04

**Authors:** Ikram Ali, Haider Darain, Muhammad Idrees Khan, Maria Ishaq Khattak, Aatik Arsh

**Affiliations:** 1 Ikram Ali, BSPT, PPDPT, MSPT, PhD Institute of Physical Medicine and Rehabilitation, Khyber Medical University, Peshawar, Pakistan; 2 Haider Darain, BSPT, MSc, PhD Institute of Physical Medicine and Rehabilitation, Khyber Medical University, Peshawar, Pakistan; 3 Muhammad Idrees Khan, DPT, MSPT District Physiotherapist, Health Department, Khyber Pakhtunkhwa, Peshawar, Pakistan; 4 Maria Ishaq Khattak BDS, MSC, PhD Institute of Public Health & Social Sciences, Khyber Medical University, Peshawar, Pakistan; 5 Aatik Arsh, DPT, MSPT, PhD Institute of Physical Medicine and Rehabilitation, Khyber Medical University, Peshawar, Pakistan

**Keywords:** Endurance, Interval training, Sedentary, Submaximal

## Abstract

**Objective:**

To determine the effects of submaximal interval training on anthropometric measurements, endurance, and flexibility of lower back and hamstrings in people with sedentary lifestyle.

**Methods:**

A quasi-experimental design was employed in this study involving 20 sedentary participants (12 females and eight males mean age: 27.20±8.48. The study conducted at Physical Therapy gym Khyber Medical University Peshawar from January 2019 to June 2019. Over four weeks, participants underwent a submaximal interval training program, comprising treadmill walking or running at 70-80% of their maximum heart rate for one minute, followed by one minute of active recovery conducted three time weekly. Assessments comprised of endurance VO2 max using the Bruce Protocol, flexibility of the lower back extensors and hamstring muscles through the Young Man Christian Association (YMCA) sit and reach test, body mass index (BMI) and waist-hip ratio (WHR).

**Results:**

The submaximal interval training intervention yielded significant improvements in endurance VO2 max, (p=0.001), flexibility (p=0.001) and BMI (p=0.016). However, the waist-hip ratio did not show any significant changes, mean pre-score (0.83±0.95) remaining the same in the post-score (0.83±0.93) (p=0.64).

**Conclusion:**

The submaximal interval training intervention demonstrated a high level of tolerance, as evidenced by a remarkable adherence rate of 91% among participants. Moreover, this training approach exhibited significant enhancements in various physical parameters.

## INTRODUCTION

Sedentary behavior and physical inactivity pose significant public health concerns, as they are closely linked to the development of cardiovascular disease (CVD), diabetes, breast cancers, and premature mortality.^1^ Insufficient physical activity in individuals is associated with an elevated risk of obesity, which is further compounded by advancing age.^2^ In addition, there exists a correlation between sedentary behavior and depression, whereas engagement in physical activity (PA) is connected to a decreased likelihood of experiencing morbidity and mortality associated with a variety of chronic ailments, including Type-II diabetes, cardiovascular disease (CVD), obesity, and diverse forms of cancer.^3^

The available evidence indicates that a sedentary lifestyle has a distinct impact on both health-related outcomes and physical function, which can be addressed as separate entities.^4^ The World Health Organization (WHO) and the American College of Sports Medicine (ACSM) advocate for a minimum of 150 minutes per week of moderate-intensity physical activity or 75 minutes per week of vigorous-intensity physical activity for individuals in good health.^5^ Individuals who fail to meet the physical activity guidelines set forth by the WHO and the ACSM are classified as physically inactive.^6^

On a global scale, it is observed that a substantial proportion of individuals, specifically 31.1%, exhibit a state of physical inactivity.^7^ Furthermore, this prevalence is experiencing a notable surge across various regions of the world. According to existing literature, a substantial proportion exceeding 50% of the adult populace in industrialized nations exhibits a state of physical inactivity.^8^ Physical inactivity is a significant contributor to obesity and mortality, the WHO 2016 report suggested that over 1.9 billion adults (39% of those aged 18 and above) were overweight, with 650 million (13%) being obese; 41 million children under the age of five were classified as overweight or obese.^9^ In developing nations, there has been a consistent decline in the prevalence of underweight individuals, accompanied by a simultaneous rise in the prevalence of obesity, as documented in various studies.^10^ Obesity presents a significant financial burden on individuals, families, and nations. In 2014, the worldwide economic ramifications of obesity amounted to US $2 trillion, in addition to its indirect costs associated with decreased productivity and absenteeism from work.^11^

The prevalence of obesity has been found to contribute to a range of health care costs, comprising between 0.7% and 2.8% of a nation’s total expenditure, the medical expenses incurred by individuals classified as obese are estimated to be 30% greater than those of individuals with a normal body mass index. The escalation of medical expenses associated with obesity in the United States (US) has been significant, with costs rising from $78.5 billion in 1998 to $147 billion in 2008; if current obesity trends persist, projections indicate that these costs could potentially reach a staggering range of $681 billion to $957 billion by the year 2030.^10^

Prolonged sedentary behavior is associated with hamstring muscle shortening and lower back muscle weakening, contributing to postural imbalances, reduced flexibility, and an increased risk of musculoskeletal discomfort.^12^ Engaging in regular aerobic exercise may help mitigate these effects by improving muscle flexibility and strength

HIIT is a versatile training method with wide-ranging applications, high-intensity exercise has demonstrated significant enhancements in performance and health outcomes among obese individuals.^13^ In sedentary and special populations, such as those with cardiovascular or metabolic diseases, HIIT is frequently employed and prescribed based on heart rate and oxygen uptake responses during incremental exercise testing.^14^ The increasing focus on promoting exercise prescription for the young and elderly population has raised concerns regarding optimizing health benefits and adherence to exercise programs.^15^

Generally, it is reported that HIIT caused high metabolic stress and has been considered high risk for adverse events in older and deconditioned adults.^16^ HIIT cause fatigue and some injuries particularly in high rate of knee and ankle sprain specially in untrained or physical inactive individuals.^17^ Moreover, it is likely to evoke negative affect and may lead to subsequent avoidance of further exercise, poor adherence and diminished self-motivation.^18^ Adherence to exercise prescription is found greater with moderate intensity exercise in sedentary people.^19^ As per our knowledge, no study has investigated the effects of submaximal interval training on anthropometric measurements, endurance, and flexibility of lower back and hamstrings in healthy individuals with a sedentary lifestyle. We hypothesized that the supra threshold intensity of the HIIT is intolerable which could diminish self-motivations required to continue the HIIT. Therefore, this causes poor adherence to regular physical activity in regard to physically inactive or sedentary population. Therefore, the submaximal interval training would be an alternative exercise regimen that may improve health outcomes in obese and people with sedentary life style.

## METHODS

A quasi-experimental design study was conducted in the Physical Therapy gym of Khyber Medical University (KMU) Peshawar, Pakistan from January 2019 to June 2019.

### Ethical Approval:

The study was approval by the ethical committee of Khyber Medical University (Study reference No. Dir/KMU-EB/SI/000603, dated May 7, 2019).

The study employed a quasi-experimental design with a single group, incorporating pre and post-test measures. The sampling technique utilized was nonprobability consecutive sampling. A total of 22 participants were recruited for the study, with 20 successfully completing their sessions. Two participants dropped out due to transportation unavailability and adverse weather conditions during their designated time period.

### Inclusion criteria:

Individuals who were physically inactive according to the guidelines set forth by the American College of Sports Medicine (ACSM), specifically those who did not engage in vigorous to moderate physical activity for 75-150 minutes per week. Additionally, participants were required to be between the ages of 20-55, have a BMI≥ 18.5, and be in good health without any cardiovascular disease or related comorbidities such as diabetes mellitus, thyroid dysfunction, or cancer and not taking any medication from the last 3 months. Participants who tested positive for risk on the Physical Activity Readiness Questionnaire (PAR-Q), had cardiovascular disease or related morbidities and had a BMI less than 18.5 were excluded from the study. The PAR-Q questionnaire is an international renowned pre participation screening tool. Many of the exercise physiologists and fitness professionals use this tool very extensively for individuals who want to participate in any regular physical activity program.^20^

Advertisements related to the study were circulated in the local community and potential participants were guided to contact research team if they are willing to participate in the study. Potential participants, who contacted research team were provided with a participant information sheet and informed consent, after re-confirming eligibility criteria. Written informed consent was obtained from eligible participants. Baseline variables were assessed a day prior to the intervention, which included endurance (VO2 max) measured by the Bruce Protocol, anthropometric measurements of BMI and waist-hip ratio (WHR), and flexibility of the lower back and hamstring muscles measured by the YMCA sit and reach test. The use of these parameters have been documented in previous literatures.^21^,^22^ Post-test was conducted a day after the intervention to ensure the effects of the last session.^23^ The intervention involving brisk treadmill walking or running at 70-80% of their maximum heart rate for one minute, followed by one minute of active recovery period. This cycle was repeated for the total of six repetitions per session, three time weekly over a month. A physiotherapist trained in treadmill-based interventions implemented this protocol.

All participants were advised to maintain their routine lifestyle throughout the study to reduce the possible confounding effects of changes in diet and routine daily physical activity. The data was analyzed using SPSS version 20. Interventions were labeled as “independent variables,” while BMI, WHR, VO2 max, and measurements from the YMCA sit and reach test were taken as “dependent variables.” Frequency, mean, and percentage were calculated for demographic information such as age, gender, and profession. Additionally, mean age was compared among the groups (male and female population). The Paired-Samples T-test was used to analyze pre and post-test differences of the numerical variables of body mass in kg, BMI, WHR, VO2 max, and YMCA sit and reach tests. Furthermore, the independent t-test was used to compare means among the groups (male and female) for significant differences. A p-value less than 0.05 was considered significant.

## RESULTS

The present study involved the calculation of results obtained from a sample of 20 participants, although, a total of 22 participants were recruited. Out of the total participants, two were not able to complete the protocol. The mean age of the participants was 27.20 ± 8.48 years, with the minimum age being 19 years and the maximum age being 43 years. Among the total participants, 40% (n=8) were male and 60% (n=12) were female population. Demographic details are presented in Table-I.

**Table-I:** Demographic Details of the participants.

	Mean± SD	Minimum age	Maximum age	Number	%
Age of patient(years)	27.20±8.48	19	43		
*Gender* Male Female				8	40.00
				12	60.00
*Profession* Student Government Employee				14	70.00
				06	30.00

The results of the post test conducted on all participants one day after the interventions are presented in Table-II. The findings indicate that there were statistically significant differences observed in various variables. Specifically, there was a significant difference in body mass, measured in kilograms (P=0.04), body mass index (BMI) (P=0.016), maximum oxygen consumption (VO2 max) (P=0.001), and the YMCA sit and reach test (P=0.001). However, it is worth noting that no statistically significant difference was observed in waist-hip ratio (WHR) (P=0.6).

**Table-II:** Paired t test analysis of the variables.

Variables	Pre test	Std	Post Test	Std	Sig.(2-tailed)
Body Mass in Kg	70.97	±11.54	70.60	±11.69	0.04
BMI	26.1	±4.50	25.9	±4.49	0.016
WHR in Inches	0.83	±0.095	0.83	±0.093	0.643
VO2 max ml/kg	30.92	±6.3	35.34	±6.4	0.001
YMCA sit& reach test in inches	18.21	±3.73	19.22	±3.85	0.001

The present study also examined the outcome measures in relation to gender. An independent sample t-test was conducted to assess the significance of differences between males and females. The results revealed that there were no significant differences between the two groups in terms of post BMI (p=0.9) and YMCA sit and reach test (p=0.4). However, significant differences were observed in post WHR (p=0.02) and VO2 max (p=0.03) between males and females. These findings are summarized in Table-III.

**Table-III:** Post Independent t test for gender comparison.

Variables	Gender	N	Mean	Std. Deviation	P-value
Post Body Mass Index	M	8	25.8462	±2.75	0.9
	F	12	26.0917	±5.47	
Post waist Hip Ratio	M	8	.8913	±.075	0.02
	F	12	.7975	±.086	
PostVO2 max in ml/kg/min	M	8	40.1725	±4.93	0.03
	F	12	32.1275	±5.26	
Post YMCA sit & reach test	M	8	20.1250	±4.47	0.4
	F	12	18.6250	3.45678	

## DISCUSSION

The present study aimed to evaluate the impact of interval training at a moderately reduced intensity level of 70-80% of maximum heart rate (MHR), referred to as submaximal intensity. The sample population consisted of 20 sedentary individuals, comprising eight males and 12 females, with an age range spanning from 20 to 55 years. Based on the findings of the study, the submaximal interval training intervention demonstrated a high level of tolerance among sedentary individuals who were overweight, with an adherence rate of 91%. This approach appears to be both feasible and effective for this specific population and holds significant implications for public health promotion efforts. Notably, the results yielded statistically significant improvements in BMI, VO2 max, and YMCA sit and reach scores, while no significant changes were observed in waist-to-hip ratio (WHR). These findings align with previous research indicating that sedentary individuals can experience improvements in aerobic fitness (VO2 max) and other health outcomes through regular exercise, particularly when exercising at intensities equivalent to at least 50% of maximum heart rate (MHR) and 50% of their individual VO2 max, as compared to physically active or athletic individuals.^24^,^25^

In a study conducted by Metcalfe RS et al. (2011), a randomized controlled trial was carried out on a sample of 29 sedentary individuals, the effects of reduced exertion high interval training (REHIT) on the participants’ VO2 max levels, comparing them to a controlled group; the results indicated a statistically significant increase in VO2 max (p= 0.01) among the REHIT group, and reported a high mean adherence rate of 97% among all the participants.^26^ These findings are consistent with those of Timothy S. Church et al. (2007), who conducted a randomized controlled trial involving 464 sedentary overweight and obese post-menopausal women. The authors of the aforementioned study concluded that the women’s overall fitness levels had improved, particularly in terms of their aerobic capacity, as evidenced by an increase in VO2 max (p=0.001).^27^

In 2013, Racil G et al. conducted a 12 weeks interval training program consisting of high and moderate intensity exercises. The study yielded noteworthy outcomes (p<.05) for body mass and BMI in both groups.^28^ These findings are in accordance with our results. Todd A. Astronio et al. (2013) reported nearly identical findings, with an adherence rate of 86% among all participants. They conducted a study on 23 sedentary women, examining the effects of two different intensity interval training levels, namely high (85-100% MHR) and moderate (75-95% MHR), on VO2 max (p=0.001). However, unlike our findings, they did not observe any changes in body composition or body weight.^29^ Notably, the study did not provide a detailed discussion on the potential reasons for the absence of these changes. Dejan Reljic et al. (2018) reported a comparable discovery regarding aerobic capacity when comparing High-Intensity Interval Training (HIIT) at 75-95% of Maximum Heart Rate (MHR) with Moderate-Intensity Continuous Training (MICT) at 65-75% of MHR in a sedentary population. Both approaches yielded significant results (p=0.001) (p=0.05) respectively, however, HIIT demonstrated superior time efficiency.^30^

The study conducted by Zhaowei Kong et al. (2016) on women with a sedentary lifestyle yielded results that were identical in nature.^31^ In a similar light, the study conducted by Amy Clark et al. (2019) examined the effects of different interval training regimens on a group of sedentary women.^32^ The results revealed a significant improvement in aerobic capacity, as measured by VO2 max (p=0.045).^32^ However, no significant changes were observed in body mass, body composition, and waist to hip ratio (p>0.05). These outcomes align with our study findings, which also demonstrated significant effects on VO2 max and not significant effects on waist to hip ratio while body mass and body composition remained unaffected. A possible explanation for this outcome is the inclusion of a control group in their study, which may have influenced the observed changes. The benefits of Physical activity has been reported in both healthy, obese and even pregnant women.^33^ The sedentary and obese population exhibits cardiopulmonary fitness levels that are approximately 50% of the recommended intensity, which is equivalent to 500 k cal/week or 75 minutes of moderate exercise per week.^28^

The present study yielded comparable outcomes with regards to cardiopulmonary fitness in sedentary overweight population. In a study conducted by Shelly E. Keating et al. (2014), obese adults were subjected to high-intensity interval training (HIIT) and moderate intensity continuous training, resulting in noteworthy enhancements in work capacity, aerobic fitness, and body composition. These findings are in line with the current investigation.^34^ Lunt et al. (2012) conducted a study comparing the effects of maximum volitional interval training (MIVT), high-intensity interval training (HIIT), and a walking group maintaining 65-75% of maximum heart rate. The study found improvements in body mass, body fat, and waist circumference, although these results were not statistically significant.

However, HIIT significantly increased cardiopulmonary endurance, which contrasts with the findings on body mass but aligns with those on waist circumference and Vo2 max^35^. The potential factors contributing to inconsistent outcomes may include a significant number of participants discontinuing the study and displaying inadequate compliance with both high-intensity interval training (HIIT) and moderate-intensity continuous training (MIVT). Nonetheless, the adherence rate of this investigation reached 91% among all subjects, indicating a high level of acceptance and practicability for sedentary individuals, including those who are overweight or obese. Consequently, this intervention could be considered an efficacious strategy for implementing routine physical activity initiatives and enhancing the well-being of individuals leading sedentary lives.

### Limitations:

The limited sample size and absence of a controlled group necessitate further investigation to ascertain the efficacy of the intervention in a larger population. Due to time constraints and limited resources, we were unable to conduct a formal sample size calculation. Future research should include a controlled group or other comparable interventions to provide a more comprehensive understanding of its effectiveness.

## CONCLUSION

The four weeks submaximal interval training particularly for sedentary individual elicited significant improvement in physical parameters including body mass index, body weight, endurance and flexibility without adverse effects. This approach yielded important implication for public health promotion. Moreover, the submaximal interval training intervention demonstrated a high level of tolerance, as evidenced by a remarkable adherence rate of 91% among participants

### Author’s Contribution:

**IA, HD, MIK, MIK** and **AA:** Conception.

**IA, HD** and **AA:** Design.

**IA, HD, MIK** and **AA:** Analysis of data.

**IA, HD, MIK** and **AA:** Interpretation.

**IA, HD, MIK, MIK** and **AA:** Critical Review. Prepared the final manuscript

All authors have approved the final version and are accountable for the integrity of the study.
